# Supplementary material for: Association of Calcitriol Supplementation with Reduced COVID-19 Mortality in Patients with Chronic Kidney Disease: A Population-Based Study
Source: Biomedicines. 2021 May 5;9(5):509. doi: 10.3390/biomedicines9050509 (PMC8147982; doi:10.3390/biomedicines9050509)
Supplement: Supplementary file 1 [file biomedicines-09-00509-s001.zip › biomedicines-1191522-supplementary.pdf]

Supplementary Tables:

Table S1. ICD-10 codes used to define SARS-CoV2 infection, comorbidities and procedures.

ICD-10 codes for clinical diagnosis of SARS-CoV2 infection: B342, B9721, B9729, J1281, J1289
ICD-10 codes for analyzed comorbidities:<br>- Arteriopathy (peripheral): E105.1, E105.2, E105.9, E115.1, E115.2, E115.9, I70.2, I70.3, I70.4, I70.5, I70.6, I70.7, I70.91, I70.92, I73.9, I96<br>- Asthma: J45, J98.01<br>- Cerebrovascular disease: G45, G46, I63, I65, I66, I672, I673, I6781, I6782, I6783, I6784, I679, M4702<br>- Cigarette smoking: F17, T65.294, Z71.6, Z72.0<br>- Chronic obstructive pulmonary disease: J41, J42, J43, J44, J47, J98.3<br>- Dementia or delirium: F01, F02, F03, F04, F05, F06, F07, F09, G30, G31, G92, G934, I674<br>- Diabetes: E08, E09, E10, E11, E13, O24, R73, O99.81<br>- Dyslipidemia: E78<br>- Heart failure: I09.81, I50, J81, I42, I43, I51.5, Z95.811, Z95.812<br>- Hypertension: I10, I11, I12, I13, I15, I16, R030<br>- Ischemic heart disease: I20, I21, I22, I23, I24, I25, Z951, Z955, Z9861<br>- Liver cirrhosis: I85, K70.2, K70.3, K70.4, K70.9, K72, K74, K76.5, K76.6, K76.7, K76.81<br>- Neoplasia (malignant): any ICD10 code beginning by C.<br>- Obesity: E66, R63.5, Z68.4<br>- Osteomalacia, rachitism and vitamin D deficiency: M830, M831, M832, M833, M835, M838, M839, E550, E559.<br>- Osteoporosis: M80, M81<br>- Parathyroid disease: E200, E201, E208, E209, E210, E211, E212, E213, E214, E215<br>- Renal failure: N17, N18, N19, Z99.2
ICD-10 codes for analyzed procedures that were performed during hospitalization:<br>- Mechanical ventilation: 5A1935Z, 5A1955Z<br>- Non-invasive mechanical ventilation: 5A093, 5A094, 5A095<br>- Orotracheal intubation: 0BH17EZ<br>- Tracheostomy: 0B9100Z, 0B110F, 0B110Z, 0B113F, 0B113Z, 0B114F, 0B114Z.

Table S2. ATC codes analyzed to identify drug use:

-Angiotensin converting enzyme inhibitors: ATC groups C09A or C09B
-Angiotensin II receptor blockers: ATC groups C09C or C09D
-Antineoplastic and immunosuppressive agents: ATC groups L01 or L04
-Dipeptidyl peptidase-4 inhibitors: ATC group A10BH
-Hydoxymethylglutaryl-Coenzyme A reductase inhibitors: ATC group C10AA
-Proton pump inhibitors: ATC group A02BC
-Systemic corticosteroids: ATC group H02

Table S3. Clinical characteristics of the patients on calcitriol treatment and matched controls

Variables	Calcitriol treated (n=6,252)	Calcitriol-matched (n=12,504)	p[1]	SMD[2]
**Variables used for matching:**				
Female gender, n(%)	3596 (57.5)	7185 (57.5)	0.954	0.001
Age, mean (SD)	70.2 (15.6)	70.7 (14.7)	0.022	0.035
Cigarette smoking, n(%)	1762 (28.2)	3562 (28.5)	0.676	0.007
Nursing home residence, n(%)	136 (2.2)	270 (2.2)	0.986	0.001
Comorbidities:				
Hypertension, n(%)	4308 (68.9)	8686 (69.5)	0.443	0.012
Obesity, n(%)	2637 (42.2)	5430 (43.4)	0.107	0.025
Diabetes, n(%)	2355 (37.7)	4857 (38.8)	0.123	0.024
Heart failure, n(%)	1691 (27.0)	3633 (29.1)	0.004	0.045
COPD, n(%)	1255 (20.1)	2638 (21.1)	0.107	0.025
Asthma, n(%)	581 (9.3)	1228 (9.8)	0.259	0.018
Chronic kidney disease, stages 3-5, n(%)	4268 (68.3)	8571 (68.5)	0.710	0.006
eGFR (mean (SD)	49.0 (30.8)	48.7 (26.6)	0.379	0.013
Cerebrovascular disease, n(%)	696 (11.1)	1487 (11.9)	0.132	0.024
Dementia, n(%)	345 (5.5)	744 (6.0)	0.246	0.019
Malignant neoplasia, n(%)	2451 (39.2)	5215 (41.7)	0.001	0.051
Liver cirrhosis, n(%)	106 (1.7)	235 (1.9)	0.406	0.014
Osteoporosis, n(%)	588 (9.4)	1168 (9.3)	0.908	0.002
Dyslipidemia, n(%)	3202 (51.2)	6406 (51.2)	0.906	<0.001
Ischemic heart disease, n(%)	963 (15.4)	2072 (16.6)	0.043	0.032
Peripheral arteriopathy, n(%)	496 (7.9)	1029 (8.2)	0.502	0.011
Drug therapies:				
Use of proton pump inhibidors, n(%)	3474 (55.6)	7094 (56.7)	0.133	0.024
Use of oral corticosteroids, n(%)	935 (15.0)	1792 (14.3)	0.262	0.018
Use of DPP4-inhibitors, n(%)	657 (10.5)	1447 (11.6)	0.031	0.034
Use of statins, n(%)	2942 (47.1)	5937 (47.5)	0.594	0.008
Use of ACE inhibitors, n(%)	1256 (20.1)	2526 (20.2)	0.872	0.003
Use of ARB, n(%)	1549 (24.8)	3143 (25.1)	0.604	0.008
Use of immunosuppressants, n(%)	602 (9.6)	1067 (8.5)	0.014	0.038
**Unmatched variables:**				
Hypoparathyroidism, n(%)	1124 (18.0)	46 (0.4)	<0.001	0.641
Renal replacement therapy, n(%)	252 (4.0)	838 (6.7)	<0.001	0.119
Functionning renal transplant, n(%)	405 (6.5)	529 (4.2)	<0.001	0.100

[1]*chi-square* (dichotomous variables) or *Student's t* test (continuous variables). [2]Standardized mean difference.

COPD: chronic obstructive pulmonary disease. eGFR: estimated glomerular filtration rate. SD: Standard deviation. ACE: angiotensin-converting-enzyme. ARB: angiotensin-II receptor blockers. DPP4: dipeptidyl peptidase-4.
